# Variability in clinical triggers for organ donation referrals

**DOI:** 10.3389/frtra.2026.1701648

**Published:** 2026-03-09

**Authors:** Kylie Casey, Elizabeth Thomas

**Affiliations:** 1 Baptist Health System, San Antonio, TX, United States; 2 UT Health San Antonio, San Antonio, TX, United States

**Keywords:** brainstem reflexes, clinical triggers, donor referral, GCS, healthcare quality, OPO, organ procurement, standardization

## Abstract

In organ transplantation, regulatory efforts have mainly targeted Organ Procurement Organizations (OPOs) and transplant centers, while donor hospitals—crucial to the donation process—have remained under-examined. As the first point of contact for potential donors, these hospitals lack standardized criteria for when and how to refer patients to OPOs, creating variability that can delay referral and reduce organ availability. This viewpoint focuses on clinical triggers: the physiological criteria that prompt hospitals to notify OPOs of potential donors. While CMS requires donor hospitals to maintain written agreements with their designated OPOs and to inform the OPO of deaths and “imminent deaths,” there is no national standard defining which bedside clinical criteria should prompt timely notifications; most hospitals defer to their local OPO for guidance. We analyzed clinical triggers from 55 of 56 U.S. OPOs and found marked inconsistency. Glasgow Coma Scale thresholds were used by 69.1%, and brainstem reflexes by 54.6%, with wide variation in both. Fewer than half addressed family discussions, and notification windows ranged from immediate to 240 min. These discrepancies reflect a critical bottleneck in the donor identification process. Standardizing clinical triggers and instituting a referral-based performance metric framework may improve metrics, thereby enhancing early donor identification, reducing missed referral opportunities, enhancing organ recovery, and reducing waitlist mortality. As scrutiny of OPOs and transplant centers increases, improving donor hospital practices is essential to optimizing the transplant system.

## 1 Introduction

The U.S. transplant system is a highly regulated, performance-driven network comprising donors, donor hospitals, Organ Procurement Organizations (OPOs), and transplant centers. Regulatory oversight is provided by the Health Resources and Services Administration (HRSA), the Centers for Medicare & Medicaid Services (CMS), the Food and Drug Administration (FDA), and the United Network for Organ Sharing (UNOS) (1). Transplant centers are evaluated on rigorous performance metrics, including pre-transplant mortality, post-transplant graft survival, offer acceptance rates, and risk-adjusted patient outcomes. Since 2019, OPOs have been under increased scrutiny following the implementation of defined CMS performance benchmarks aimed at improving efficiency in organ procurement. Under the 2020 Notice of Proposed Rule Making (NPRM), OPOs are evaluated on their donation rate (the conversion of potential donors to actual donors) and their transplant rate (the number of organs transplanted per donor). These metrics aim to increase transplant volumes—especially kidney transplants—by 2030 (2).

Despite these reforms, many OPOs continue to lag performance benchmarks. According to the 2023 CMS OPO Performance Report, OPOs are at risk of decertification and replacement by higher-performing organizations within their donor service area (DSA) (3). Transplant centers face similar existential threats, including loss of insurance contracts, site visits, and oversight through models like IODA (Improving Outcomes in Donation and Transplantation). In contrast, donor hospitals—the critical entry point to the organ donation process—have not been included in the otherwise detailed and specific regulatory transplantation framework. Under the CMS State Operations Manual §482.45, hospitals are required to maintain written agreements with their designated OPOs and ensure timely referral of deaths and patients meeting imminent-death criteria. However, CMS survey guidance primarily focuses on verifying the existence of these agreements and general compliance with Conditions of Participation, rather than auditing the quality, timeliness, or physiologic appropriateness of donor referrals. As a result, hospitals may technically meet CMS requirements while exhibiting wide variation in referral behavior and trigger implementation.

Clinical triggers are the physiological or clinical criteria used to identify potential organ donors, serving as the initial gateway to the organ donation process (4). Despite their centrality, no national standard governs their use; thus, practices vary widely across different OPOs. This variability contributes to inconsistent referral practices, delays in donor identification, and missed opportunities for organ recovery. We aimed to characterize and compare the content of OPO-issued clinical trigger materials used to prompt donor referral, and to describe variability across OPOs that may inform standardization and future outcomes-linked evaluation.

While the focus of this paper is on clinical triggers, they serve as a representative example of the broader lack of standardization and accountability in donor hospital practices. Addressing this variability offers a meaningful opportunity to optimize early-stage organ procurement. As OPOs and transplant centers face increasing regulatory scrutiny, elevating the performance and accountability of donor hospitals is essential to bridging gaps in the transplant continuum and creating opportunities to save lives through transplantation.

## 2 Materials and methods

### 2.1 Data collection

Data were gathered from 55 of 56 OPOs in the United States through direct contact with OPOs or when OPOs did not respond to direct requests, through indirect sources such as copies of trigger materials obtained from donor-hospital staff and publicly accessible online information. Internet sources included academic presentations and hospital policies related to organ procurement and clinical triggers. We abstracted trigger criteria from the source documents and did not collect patient-level data. Donor-hospital staff served as conduits to obtain documents and were not analyzed as the “responsible party” for referral.

### 2.2 Content of clinical triggers

Clinical triggers were analyzed in terms of their inclusion of the following:

Glasgow coma scale (GCS): If this was included, the specific threshold value used to determine referral eligibility was recorded. Lower GCS scores indicate a more severe neurological impairment and a higher likelihood of brain death, making this criterion vital for assessing patient eligibility for organ donation.
Brainstem reflexes, including gag, corneal, and oculocephalic reflexes: The absence of these reflexes often indicates significant brain injury, which is crucial in determining the potential for organ recovery.
Family discussions on patient prognosis or withdrawal of care: These discussions are essential to facilitating the consent process and ensuring ethical alignment with the patients' and their families' wishes.
OPO notification time after clinical triggers have been met: This timeframe is critical because delaying the initiation of the organ donation process affects the success of organ recovery.

### 2.3 Top-performing OPOs

OPO performance was determined using the CMS 2023 interim annual public aggregated performance report, which evaluates donation rate (the proportion of potential donors who become actual donors) and transplant rate (the number of organs transplanted per donor), both adjusted for age and regional donor potential. CMS derives these metrics from linked data sources including the Scientific Registry of Transplant Recipients, National Center for Health Statistics multiple cause-of death files, Medicare inpatient claims, and county-level waiver files to estimate the potential donor pool (4). OPOs ranking in the top performance tier across both metrics were designated as top-performing. CMS proposed that OPO recertification be based on two primary outcome measures: donation rate and transplant rate. Donation rate reflects conversion of potential donors to actual donors (donors per potential donors). Transplant rate reflects the number of organs transplanted per donor (organs transplanted per donor). CMS derives these measures using linked national datasets and applies risk adjustment to support comparisons across OPOs. Analyses by the SRTR demonstrated that over half of OPOs would fail at least one of these standards under the proposed rule, underscoring the sensitivity of performance rankings to early donor identification and referral practices. The top 10 OPOs were identified as Sierra Donor Services, Nevada Donor Network, Live On Nebraska, Midwest Transplant Network, Mid-America Transplant, Life Center Organ Donor Network, UW Health Organ and Tissue Donation, Life Connection of Ohio, Gift of Life Donor Program, and Our Legacy.

### 2.4 Data analysis

Clinical trigger data collected from all OPOs were descriptively analyzed to evaluate patterns and variability. The variables examined included the frequency and specificity of GCS thresholds, brainstem reflex criteria, family discussion guidelines, and timeframes for contacting OPOs after meeting the clinical trigger criteria. The documentation included the usage period for both historical and current clinical triggers. Descriptive statistics, such as frequencies and percentages, were calculated using Microsoft Excel (Microsoft, Redmond, WA, USA) and visualized with the software's built-in tools.

### 2.5 Web tool development with clinical trigger cards

Clinical trigger cards are visual aids OPOs provide to bedside nurses, the only healthcare professionals authorized to contact an OPO based on the clinical criteria specified on the cards (5).

These cards are commonly attached to nurses' badge reels or posted around hospital units for quick reference. However, the format and distribution of these cards vary widely among hospitals, and some OPOs also have specific protocols for their use. Clinical trigger cards were collected from each OPO and analyzed, with the obtained data used to create an interactive web-based tool called “Refer my Patient” to display the clinical trigger visual aids. The website (http://www.refermypatient.us) was coded in JavaScript and hosted on the public Cloudflare platform (Cloudflare, San Francisco, CA, USA) to ensure secure access and accurate data representation.

## 3 Results

Data were successfully gathered from 55 of the 56 OPOs: 39 through contact with local nursing staff, 9 through internet searches, 4 through direct responses from OPOs, and 3 through hospital policies. Despite numerous efforts to contact LifeLink of Puerto Rico, including phone calls, emails, and outreach through their website, we could not obtain data as attempts at contact went unanswered. Additionally, no local nurses working at hospitals within the LifeLink Puerto Rico DSA could be found to provide information. Direct responses were received from 7.3% of the OPOs, while the remaining data were obtained from indirect sources. Only 9.1% of the OPOs provided information on historical clinical triggers. Information on the duration of use of current clinical triggers was available at 24 OPOs. The implementation years for current clinical triggers ranged from 2006 to 2023, with the following implementation years identified: 2006, 2010, 2013, 2017, 2019, 2020, 2021, 2022, and 2023.

The GCS was incorporated into the clinical triggers of 69.1% of OPOs. Among them, the threshold for initiating a referral to an OPO ranged from 4 to 8, with 4 used by 3.6% of OPOs, 5 used by 61.8%, and 8 used by 3.6%. The average threshold was 5.1.

Brainstem reflexes were variably included by 54.5% of OPOs, with absence of pupillary response (32.7%), absence of gag reflex (31.0%), absence of cough reflex (31.0%), absence of spontaneous respiration (18.2%), absence of response to painful stimuli (27.3%), absence of cold caloric reflex (5.5%) (6), absence of oculocephalic reflex (5.5%), and absence of corneal reflex (21.8%). Additionally, OPOs had different requirements: 27.3% needed one reflex to be absent, 20.0% needed two, and 5.5% needed three. One response noted that the loss of “certain neurological reflexes” was necessary, although no further details were provided.

Family discussions were included by 49.1% of OPOs. The description of these discussions varied, ranging from general notifications to detailed guidance on timing and content. There was also considerable variability in the time allowed to notify OPOs, ranging from 0 to 240 min. The most common time was 60 min, reported by 81.8% of OPOs, followed by 0 min, (7.3% of OPOs), 120 min (5.5%), 240 min (3.6%), and 180 min (1.8%).

### 3.1 Top-performing OPO results

Of the top 10 rated OPOs identified by the CMS, 70% included the GCS as a clinical trigger; only 50% mentioned brainstem reflexes. Four OPOs specified that at least one reflex must be absent, while one OPO specified that two reflexes must be absent. The specific reflexes also varied among these OPOs; two required the absence of pupillary responses, gag reflexes, and cough reflexes, whereas three referred to the lack of “some neurological reflexes” without further specification. No two OPOs used the same criteria for brainstem reflex assessment.

**Figure 1:**
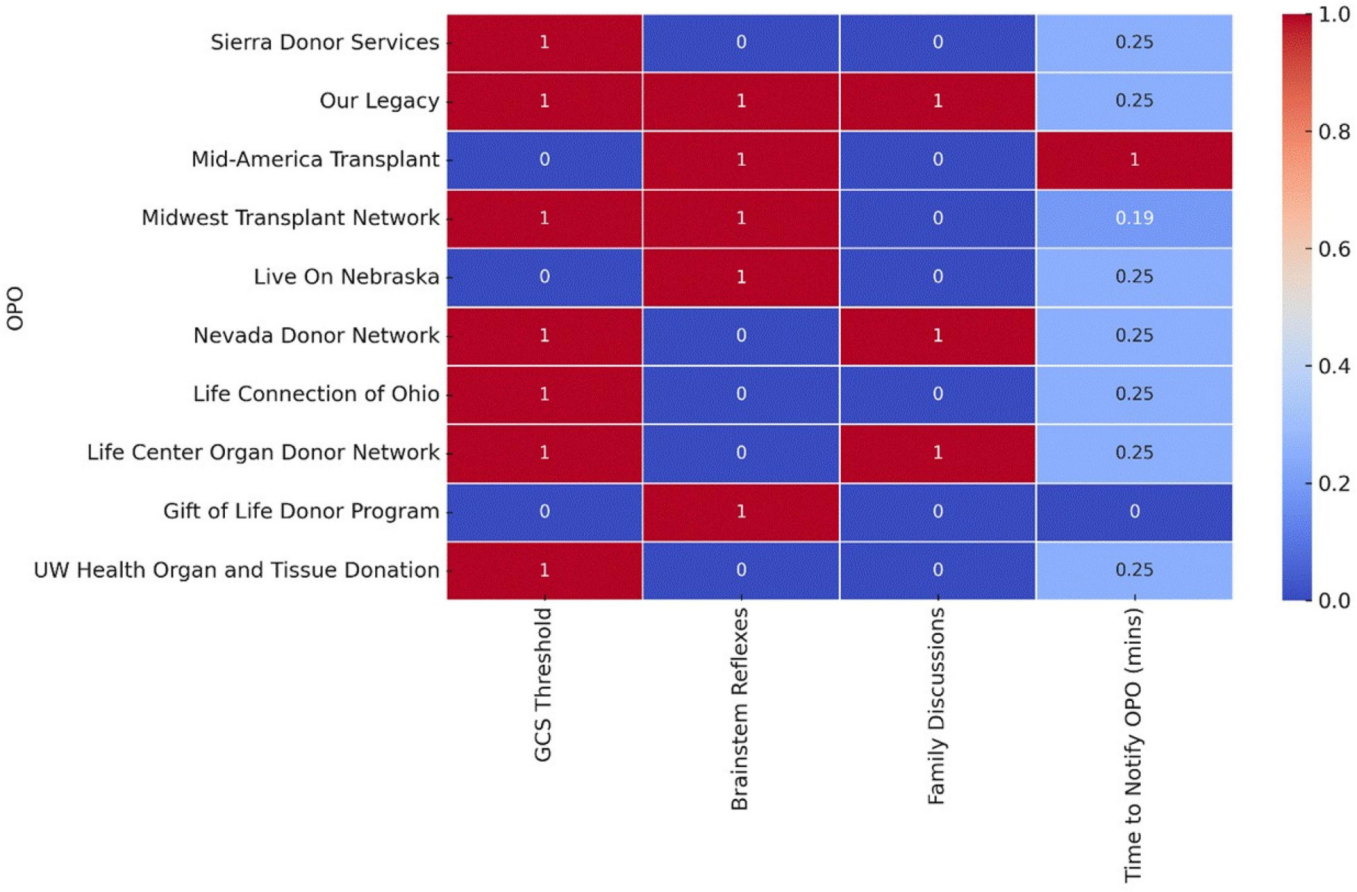
Variability in clinical triggers among the top 10 rated OPOs. The heatmap displays the presence and variability of key clinical triggers used by the top 10 OPOs. Red indicates the presence of a specific clinical trigger (e.g., a defined GCS threshold, brainstem reflexes, or family discussions), while blue represents its absence. For the “Time to Notify OPO (mins)” category, the color gradient from blue to red reflects a normalized scale, with darker red indicating shorter notification times and darker blue representing longer times.

Among the top 10 OPOs, 30% explicitly mentioned family discussions in their clinical triggers, with varying approaches. Only one OPO issued specific guidance on the timing of family discussions. The notification timeframes were inconsistent among the OPOs: 70% of the top OPOs specified 60 min, one allowed a 30–60-minute window, and another set it at 180 min. Figure 1 illustrates the differences among the highest-performing OPOs regarding the inclusion of GCS thresholds, brainstem reflex assessments, family discussion protocols, and time-to-notification requirements.

Under CMS's proposed framework, the national donation rate averaged 3.59 donors per 100 potential donors, yet top-performing OPOs exceeded 6 donors per 100 potential donors while the lowest performers fell below 2 donors per 100 potential donors. Similarly, organ transplant rates varied more than four-fold across DSAs. This performance dispersion suggests that even modest improvements in early donor identification and referral standardization could translate into substantial national gains in transplantable organs.

### 3.2 Interactive map with results

**Figure 2:**
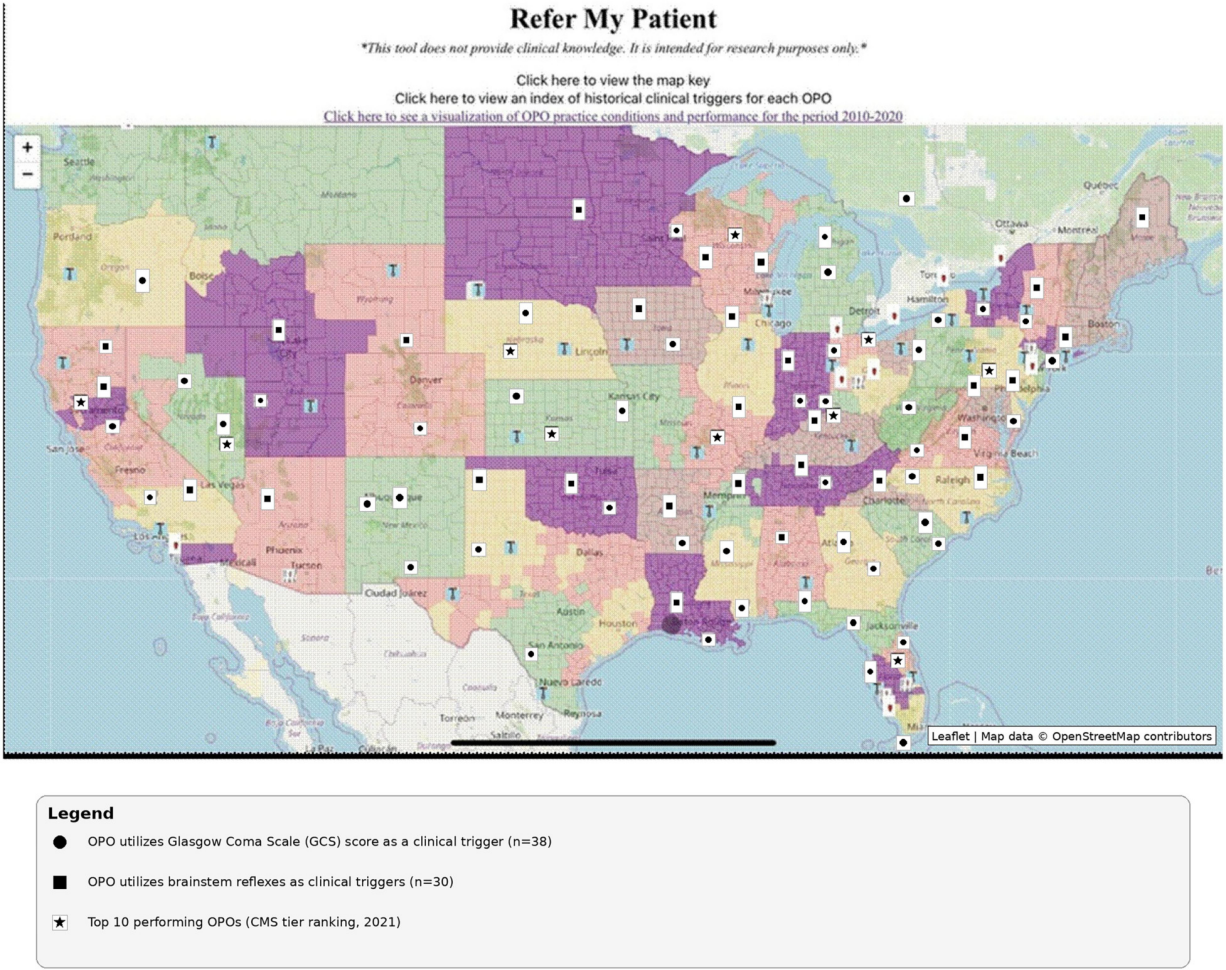
Interactive map of organ procurement organizations across the United States, showing the clinical triggers issued by each OPO, with map key. Map adapted from OpenStreetMap (https://www.openstreetmap.org/), licensed under Open Data Commons Open Database License (ODbL) v1.0. Adapted with permission from plotly/datasets (“geojson-counties-fips.json”), licensed under MIT License.

Figure 2 shows an interactive map from the website (http://www.refermypatient.us), which was developed to visually represent the geographical distribution of OPOs across the United States and the map key. The map key explains the meanings of the symbols and color codes used in the map and enables viewers to identify OPOs that use GCS scores and brainstem reflexes as part of their clinical triggers. The OPOs ranked in the top 10 by the CMS are highlighted.

**Figure 3:**
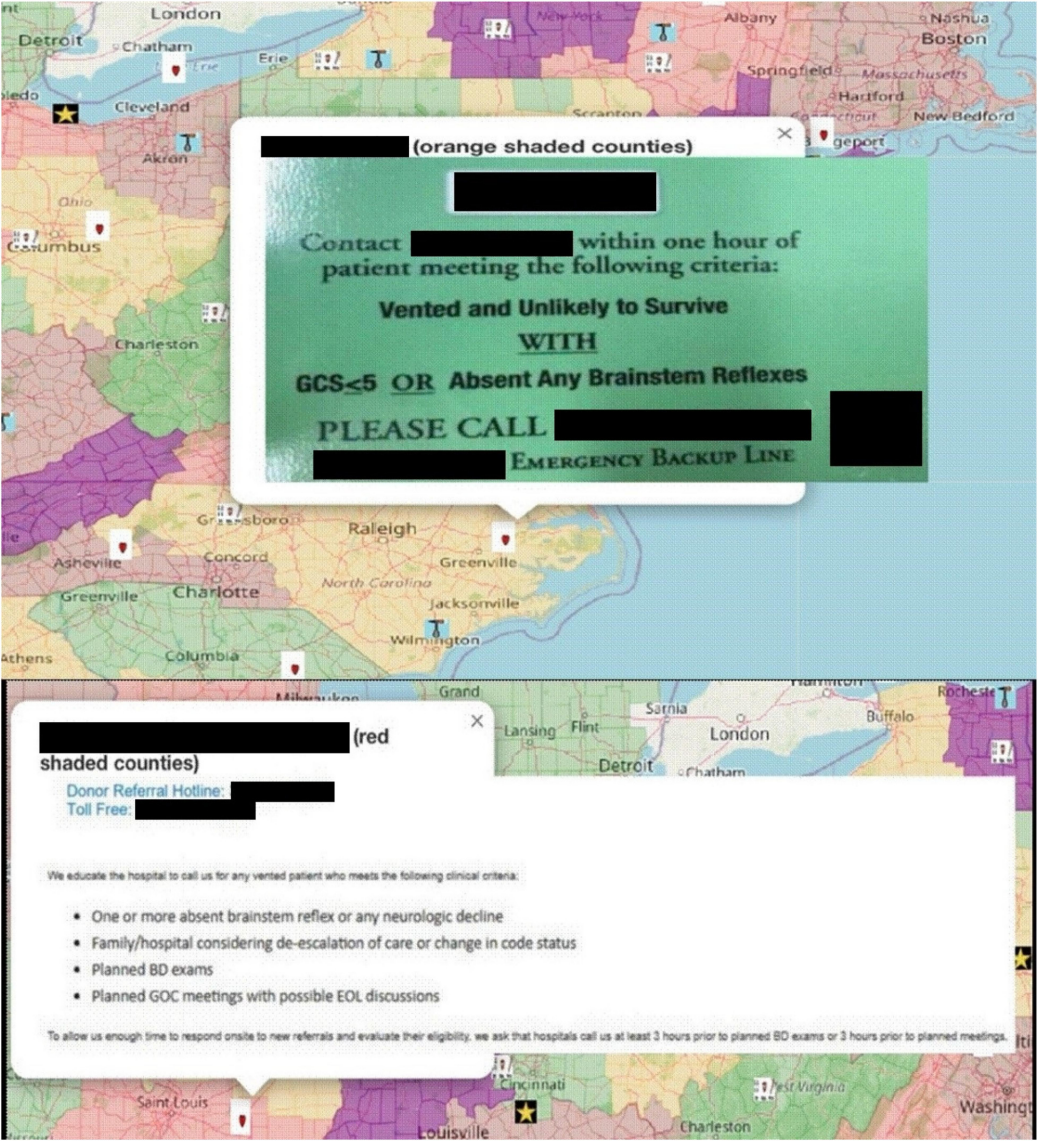
Examples of clinical trigger cards. Maps adapted from OpenStreetMap (https://www.openstreetmap.org/), licensed under Open Data Commons Open Database License (ODbL) v1.0.

Clinical trigger cards, with examples shown in Figure 3, were included in the interactive map.

When a particular clinical trigger card was unavailable for analysis, its absence was noted on the map, and the indirect source data was included instead.

## 4 Discussion

Our analysis of clinical triggers revealed substantial variability in referral practices, highlighting a lack of standardization in donor eligibility criteria. Key indicators, such as GCS thresholds and brainstem reflex assessments, were applied inconsistently across OPOs. GCS thresholds ranged from 4 to 8, with 5 being the most common. Brainstem reflex criteria varied widely, with some OPOs grouping them under vague terms like “neurological reflexes.” These inconsistencies reflect broader underlying concerns about donor hospital referral practices. Although clinical triggers are defined by OPOs, donor hospital staff use them as the first decision point in the donation process. Because hospitals are expected to notify the OPO of deaths and imminent deaths, trigger tools should be interpreted as operational prompts that support timely notification—not a substitute for the hospital's duty to notify. This analysis uses clinical triggers as a case example of how upstream variability may affect downstream donor availability and organ recovery outcomes. The absence of uniform referral standards introduces inefficiencies and delays in donor identification and notification, potentially contributing to missed referrals and ultimately reducing donor conversion and transplant availability. A 2018 systematic review by Squires et al. evaluated 124 studies and identified four core categories of clinical criteria for deceased donor identification: neurologic, medical-decision, cardiorespiratory, and administrative triggers. The authors emphasized that the absence of a universally accepted set of clinical triggers contributes to missed donor opportunities and confusion among critical care clinicians responsible for initiating the referral process, concluding that consistent application of neurologic decline-based criteria could reduce ambiguity and improve conversion of potential to actual donors (7). Reflex-based criteria may be particularly vulnerable to misinterpretation. Henry et al. demonstrated that ventilator auto-triggering can falsely appear as spontaneous respiration in patients with absent brainstem reflexes, delaying brain-death determination and potentially leading to loss of donation opportunities. In a single OPO review of 672 referrals, 63 cases were identified in which ventilator auto-triggering obscured the absence of intrinsic respiratory drive, delaying progression to donor evaluation (8). These findings support the preferential use of standardized neurologic decline triggers, such as GCS thresholds, rather than reflex-based assessments alone.

Among the clinical triggers analyzed, inclusion of a defined GCS threshold—particularly scores ≤5—was the most consistently used indicator across both all OPOs (69.1%) and top-performing OPOs (70%). In contrast, brainstem reflex criteria and family discussion triggers were less consistently applied, even among high-performing organizations. These findings suggest that a low GCS threshold may serve as the most reliable and actionable physiologic trigger for early donor identification. Prior analyses of high-performing OPOs similarly report that early neurologic decline, rather than reflex-based assessments, is more strongly associated with timely donor referral and conversion, supporting GCS-based triggers as a practical focal point for standardization. However, causal relationships cannot be inferred from these observational data. CMS performance stratification has demonstrated more than two-fold variability in donation and transplant rates between top- and bottom-quartile OPOs nationally (3). This magnitude of difference underscores the potential impact that systematic improvements in early referral practices—such as standardized clinical triggers—may have on national organ availability and transplant access.

While transplant centers and OPOs are held to rigorous performance metrics, donor hospitals lack comparable standards—despite serving as the entry point to the donation process. This disparity in accountability has been flagged by national groups, including the Organ Transplantation Advisory Group and the National Academies of Sciences, Engineering, and Medicine, both of which call for improved coordination, transparency, and standardization across the transplant ecosystem (9). Similarly, the Organ Donation and Transplantation Alliance has emphasized the importance of establishing clear referral pathways and performance metrics for donor hospitals (10). Our findings reinforce these recommendations by demonstrating that clinical trigger variability reflects a larger oversight failure in early-stage referral practices (11). While previous studies have addressed donor identification more broadly, this is among the first to map clinical trigger criteria across U.S. OPOs systematically. These inconsistencies—particularly in GCS thresholds and reflex definitions—signal a fragmented, variably implemented referral process. Without better alignment between early hospital practices and downstream performance expectations, efforts to improve national organ recovery efficiency may fall short.

In addition to standardizing clinical criteria, reevaluating the structural and contractual relationships between OPOs and donor hospitals may improve cooperation and performance. OPO–hospital cooperation contracts are often the only formal mechanism for communicating expectations. Stronger language in these agreements—such as clearly defined clinical triggers, response times, and communication protocols—could help enforce accountability and encourage earlier referral behaviors. However, because this study did not link trigger content to referral volume or donation outcomes, any “high-yield” trigger set should be considered hypothesis-generating and tested using outcomes-linked evaluation. Comparing contract content across top-performing and lower-performing OPOs may reveal common strategies that correlate with higher donor conversion rates. Furthermore, aligning hospital incentives with donation goals could address a core disincentive for timely referral: concern over mortality statistics. One policy concept discussed in the field is the exclusion of registered or referred donors from a hospital's publicly reported mortality metrics. This may reduce clinical teams' hesitation to refer eligible donors, particularly in high-acuity cases. Other incentive structures—such as recognition programs, financial alignment, or regulatory credits warrant exploration. Prioritizing positive reinforcement over punitive oversight could foster stronger collaboration and increase organ availability.

Despite its contributions, this study has limitations. The majority of clinical trigger data were obtained indirectly through local nursing staff, public websites, or hospital policies due to a low rate of direct OPO responses. As such, the findings may not reflect real-time updates or local nuances. Additionally, the observational nature of this study precludes causal conclusions regarding the relationship between clinical trigger variability and donor conversion outcomes. Confounding factors—such as regional differences in staffing models, hospital size, or OPO–hospital relationships—were not examined in this analysis but may influence referral behaviors and outcomes. Despite this, the study's strength lies in its scope; to our knowledge, it is one of the first comprehensive national evaluations of clinical trigger variability across nearly all U.S. OPOs. The implications of standardizing clinical triggers extend well beyond improving early referral processes. Inconsistent referral practices may directly influence downstream performance metrics, such as Cause, Age, and Location Consistent (CALC) deaths—a retrospective CMS measure used to assess missed donor opportunities. These missed opportunities are often attributed to inefficiencies in the early stages of the donation process.

Implementation of standardized, evidence-based clinical triggers—such as uniform GCS thresholds, clearly defined brainstem reflex criteria, and consistent OPO notification timeframes—may help healthcare systems better align referral practices with CMS's outcome-driven metrics. The referral timeline from neurologic decline to OPO notification remains opaque in most donor hospitals. Bedside clinicians often rely on informal heuristics rather than structured triggers, delaying engagement with OPOs until after withdrawal-of-care discussions or catastrophic neurologic events. This delay may compress donor management windows, compromise organ quality, and reduce conversion opportunities. Standardized early-stage triggers—particularly neurologic decline thresholds—could shift referral upstream, allowing OPO teams to engage earlier in donor optimization and family support. It is noteworthy that even among top-performing OPOs, there was no consistent set of clinical triggers, indicating a disconnect between how performance is measured and how early-stage referral decisions are operationalized. Automated donor referral systems may mitigate delays inherent to manual identification processes. In a multi-hospital study evaluating automated referral deployment, Levan et al. demonstrated a 45% increase in ventilated referrals, an 83% increase in approaches for authorization, and a 92% increase in actual organ donors following implementation of electronic trigger-based referral technology (12, 13). These findings highlight that the referral timeline is not merely a clinical workflow issue, but a system-design problem in which reliance on subjective bedside identification materially limits donor potential. The results of this study suggest that standardizing clinical triggers is not merely a logistical improvement, but a pivotal foundational step towards system-wide reform. Given the persistent organ shortage and rising transplant waitlist mortality, inefficiencies in donor referral pathways demand immediate attention.

To improve feasibility, we propose that standardization be approached as a staged implementation: (1) define a candidate minimum trigger-domain set based on the most commonly recurring criteria identified in this review; (2) pilot implementation within health systems with predefined process metrics (e.g., timeliness of OPO notification after triggers are met, documentation of notification, and adherence to local policy); and (3) evaluate downstream outcomes before broader adoption. Regulatory bodies, OPOs, transplant leaders, and hospital systems must collaborate to develop and implement national standards that include defined physiologic thresholds, structured family discussion guidelines, and clear time-sensitive notification policies. Additionally, incorporating process-based metrics alongside outcome-based metrics into donor hospital evaluations may provide a comprehensive view of the potential for organ recovery. Without such reforms, inconsistencies in early referral practices will continue to limit organ availability and delay life-saving transplants. Clinical triggers are a critical area where early intervention toward actionable improvements should serve as a catalyst for broader donor hospital accountability and integration into the national transplant quality framework.
