# Supplementary material for: Variability in clinical triggers for organ donation referrals
Source: Front Transplant. 2026 Mar 9;5:1701648. doi: 10.3389/frtra.2026.1701648 (PMC13007231; doi:10.3389/frtra.2026.1701648)
Supplement: Supplementary file 1 [file Datasheet1.pdf]

To review the referenced website: https://refermypatient.us

Password (case sensitive): SaveLives1
